# Kinetics of Muscle Damage Biomarkers at Moments Subsequent to a Fight in Brazilian Jiu-Jitsu Practice by Disabled Athletes

**DOI:** 10.3389/fphys.2019.01055

**Published:** 2019-08-23

**Authors:** Jaqueline Santos Silva Lopes, Aníbal Monteiro de Magalhães Neto, Luís Carlos Oliveira Gonçalves, Paulo Ricardo Lourenço Alves, Aline Castilho de Almeida, Claudia Marlise Balbinotti Andrade

**Affiliations:** ^1^Medicine Department, Postgraduate Program in Health Sciences (PPGSC), Federal University of Mato Grosso (UFTM), Cuiabá, Brazil; ^2^Department of Physical Therapy, Centro Universitário do Vale do Araguaia (UNIVAR), Barra do Garças, Brazil; ^3^Department of Physical Education, Federal University of the State of Rio de Janeiro (UNIRIO), Rio de Janeiro, Brazil; ^4^Technical School Support Foundation, Visconde de Mauá State Technical School, Rio de Janeiro, Brazil; ^5^Department of Physiotherapy, Federal University of São Carlos (UFSCAR), São Carlos, Brazil

**Keywords:** creatine kinase, martial arts, sports medicine, physical therapy specialty, inflammation, musculoskeletal physiological phenomena

## Abstract

**Purpose:** Evidence indicates that muscle injury caused by exercise can lead to functional, biochemical, and clinical damage. These outcomes encompass an intrinsic potential to understand the real magnitude of interpretation of classic signs in sport environments and to monitor athletes, contributing to specific actions. However, little or no research has explored the general behavior of the variables presented in response to paradesportivo Brazilian jiu-jitsu. The objective of this study was to investigate the physiological behavior through clinical, functional, and metabolic outcomes in the moments following a simulated fight.

**Methods:** Six disabled athletes, male Brazilian jiu-jitsu practitioners (34–44 years old), were included. The participants had their outcomes analyzed individually and the variables studied were correlated. It is noteworthy that participants I and II are professional athletes with world titles. The ethics committee involving human beings of the Federal University of Mato Grosso (register no. 2.997.241) accepted the study. The participants attended the collection site four times, with a 24-h interval between sessions, characterizing the following moments: pre-exertion, and post-exertion, 24, 48, and 72 h after the simulated fight. Data collected were muscle pain, perception of recovery, muscle strength, and blood samples for creatine kinase (CK) and lactate dehydrogenase (LDH) analysis. The variables described were measured at all collection moments. The data were presented in individual raw values of each participant, with Spearman correlation analysis to verify the relationship between variables and moments.

**Results:** The outcomes demonstrated that the CK and LDH activity was higher of high-performance parathletes (I and II) and the reported muscle pain was lower. The fight did not influence maximal isometric strength levels in either participant. In addition, regarding delayed effects, the participants reported peak pain, CK, LDH, and decreased perception of recovery within 24 h. However, it was found that, at 72 h, all values had recovered, close to baseline levels.

**Conclusion:** The presented outcomes provide parameters and suggest a safe scenario based on the intensity and volume commonly adopted in this sports parade modality where the level of effort recommended during combat does not seem to cause deleterious damage.

## Introduction

Martial arts are related to a complex set of corporal strategies that include physical and mental aspects. Brazilian Jiu-jitsu, represented by intermittent movements of high intensity interspersed by brief periods of less intensity, characterizes one form of martial art (Andreato et al., 2017). With regard to the parasport, Brazilian jiu-jitsu has been demonstrated to be an inclusive sport. As for inclusive sport, several studies (Tweedy and Vanlandewijck, 2011; Tweedy et al., 2014; Ravensbergen et al., 2016; Stoter et al., 2017) classify parathletes according to the different levels of deficiencies presented.

It is known that inclusion in sports tends to provide positive repercussions on anthropometric, physiological, social, and psychological measures (Tweedy and Vanlandewijck, 2011; Tweedy et al., 2014; Ravensbergen et al., 2016; Stoter et al., 2017). However, athletic performance is related to levels of overload, which, when not properly administered and periodized, can lead to damage to the body systems (Damas et al., 2016). These losses, considered as exercise-induced muscle damage, include loss of the capacity to generate force, reduction in range of motion, muscular pain, and edema (Buchheit et al., 2009; Chen et al., 2013; Belli et al., 2018).

When considered, clinical and functional parameters are able to diagnose the general recovery condition of the subject. In this sense, previous studies (Cesnaitiene et al., 2015; Machado et al., 2018; Papassotiriou and Nifli, 2018) have shown that these variables also have a positive correlation with athletic performance and adequate monitoring allows the use of strategies that improve recovery and sports performance and minimize exposure to the occurrence of musculoskeletal injuries.

To analyze the deleterious effects mentioned above, based on the inexistence of studies that address the outcomes mentioned in paradesports Brazilian jiu-jitsu, it is considered pertinent to investigate the physiological responses under these conditions. Moreover, the results found could contribute to the understanding of the real magnitude of the interpretation of classic signs in a sports environment, contributing to guidance on specific intervention actions.

To our knowledge, this is the first study to propose the investigation of the kinetics of muscle damage in Brazilian jiu-jitsu paradesports. We believe that the disability, characterized for example by the lack of a limb, may require higher metabolic demands and result in a greater level of muscular damage, compared to non-disabled athletes. Therefore, the objective of the present study was to investigate the immediate and delayed physiological responses triggered by a fight, in Brazilian Jiu-Jitsu parathletes with different levels of physical conditioning.

## Materials and Methods

### Participants

Six male Brazilian jiu-jitsu parathletes participated in the study. It is noteworthy that participants I and II are professional athletes with world titles. The eligibility criteria adopted included the practice of paradesportivo Brazilian jiu-jitsu for a period of more than 6 months. The sample size was characterized by a convenience scenario, attributed to the logistical difficulty in grouping a high number of participants with the inclusion characteristics adopted in the study.

For define the sample size, an *a priori* knowledge was used, based on the findings of Andreato et al. (2015). The chosen variable referred to the values of creatine kinase in subsequent moments to the simulated fight of Brazilian jiu-jitsu. For that, two-tailed hypothesis test was used, with significance level of 5 and 80% of power and possible sample loss of 15%, the stipulated sample size would correspond to eight volunteers.

All participants attended the same training center, and no musculoskeletal injuries were reported during the procedures. The anonymity of the participants was guaranteed. Masking of the participants, investigator, and evaluator was performed regarding the results, the hypotheses, and analyzed outcomes.

In addition, to be included, participants were required to report the absence of anemia, inflammation, diabetes, cardiovascular disease, and musculoskeletal injuries within 6 months prior to data collection. Furthermore, they were advised to refrain from anti-inflammatory drugs, analgesics, alcoholic beverages, and tobacco and not to perform any exercise not proposed by the study.

All subjects followed a similar diet and did not receive special supplements. Thus, at each collection session, all guidelines on a controlled diet were reinforced and participants were asked about possible diet adversity. In this sense, the diet control of the participants was performed only subjectively by verbal orientations.

For description of the functional classifications and types of injuries, the participants had physical motor disability (66.4%) and visual disability (33.6%), represented by functional classification S6 (amputations of the leg) and S12 (partial visual disability), respectively.

The anthropometric characteristics of the participants are presented in Table 1.

**Table 1:** Anthropometric characteristics of participants.

	Age (years)	Stature (m)	Body mass (kg)	Training time (years)	Training time per week (h)	Competitive level	Belts
Parathlete I	43	1.63	96.5	22	18	Professional	Black belt
Parathlete II	36	1.70	82.3	7	9	Professional	Purple belt
Parathlete III	41	1.65	90.0	1	7	Amateur	White belt
Parathlete IV	34	1.76	89.0	1.2	6	Amateur	Blue belt
Parathlete V	13	1.46	35.5	0.9	5	Amateur	Blue belt
Parathlete VI	40	1.63	84.0	0.7	6	Amateur	Blue belt

*Parathletes I and II were world champions in their respective categories*.

### Ethics Statement and Clinical Trial Registry

The participants were informed about the procedures and objectives of the study and, after agreeing, signed a consent form. The consent obtained from the participants was both informed and written. The Ethics Committee in Research of the Federal University of Mato Grosso previously approved all procedures (Araguaia campus, sob seem number: 2.997.241).

### Study Design

This is an observational study. Data collection was carried out in June 2018 at the usual training center of the participants (Grace Barra Academy) located in the municipality of Barra do Garças, MT, Brazil, and data analyses were conducted at the Federal University of Mato Grosso, Araguaia Campus. All procedures were performed under standard conditions (temperature: 28 ± 1°C, relative humidity: 84%).

**Figure 1:**
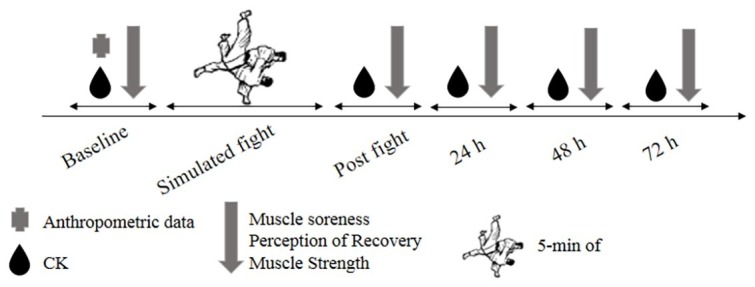
Design study. CK, creatine kinase; 5-min of, five minutes of simulated fighting.

The procedures took place on four consecutive days, always in the same period, in order to avoid influences of the circadian cycle. At first, all participants were submitted to an anthropometric evaluation (Table 1) using a scale (Tanita BC554, Iron Man/InnerScaner, Tanita, Illinois, USA) and a stadiometer (Sanny, American Medical do Brasil, São Paulo, Brazil). On the first day, a simulated fight was performed as well as evaluation of the metabolic, clinical, and functional parameters in the pre- and post-fight moments. On the subsequent days (24, 48, and 72 h after the fight), all parameters were reevaluated in order to measure outcomes related to the delayed effect. An overview of the study is presented in Figure 1.

The order of the fights was defined by prior randomization. A warm-up was performed with Brazilian jiu-jitsu movements of light intensity, characterized by low heart rate and low strength requirement, for 5 min. The simulated fight protocol occurred in accordance with the rules of the International Brazilian Jiu-Jitsu Federation (IBJJF), excluding any type of finalization (International Brazilian Jiu-jitsu Federation (IBJJF), 2018). In these cases, the parathletes were separated and directed to return to the fight immediately. Thus, maximum effort was advocated as well as a similar activity for all participants. The parathletes fought with non-disabled athletes, not included in the study, who were previously trained and guided on standardized fighting behavior, with all participants, in order to minimize the influence of possible bias. The choice of opponents was based on similar graduation similar body mass.

### Procedures

#### Blood Sampling and Analysis

Creatine kinase (CK) and lactate dehydrogenase (LDH) activity were verified by blood collection from the antecubital vein, collected using a syringe (62 μl) by a qualified professional. The blood sample, evaluated in serum, was analyzed by the ELISA method and an Advia 1650 analyzer (Siemens Healthcare Diagnostics, Deerfield, IL, USA) in a specialized laboratory.

#### Muscle Soreness

Participants were instructed to assess muscle discomfort (induced by the simulated fight) using a Visual Analogue Scale (VAS) ranging from 0 “no pain” to 10 “extreme pain” (Machado et al., 2017).

#### Perception of Recovery

The perception of recovery was obtained using a 10-point Likert Scale, where 1 indicates the feeling “not recovered” and 10 indicates “fully recovered”. The participant was asked the following standardized question: If you had to perform the fight again at this time, how recovered do you feel? (Lopes et al., 2018).

#### Maximum Voluntary Isometric Contraction

Measurement of the Hand Grip Strength (HGS) was performed using an analog hydraulic dynamometer, brand JAMAR^®^ (Asimow Engineering^®^, USA), with an accuracy of 0.5 kg/f and a maximum capacity of 100 kg/f. The position suggested by the American Society of Hand Therapists (ASHT) was used to perform the test (Franchini et al., 2011), which occurred with the participant in a sitting position, with hips and knees at 90° flexion, shoulder at adduction, elbow flexed at 90°, and wrist and forearm in a neutral position, resting on a table. The participant was instructed to use the greatest possible strength, and the peak value was subsequently recorded.

### Statistical Analysis

The raw data of each participant were presented for each investigated variable.

Spearman’s correlation was used between the analyzed outcomes (pain, recovery, strength, and muscle damage) and were compared between the post-fight, and the other moments were evaluated (baseline, 24, 48, and 72 h). The variables were considered as independent–designated as ***X***, at the post-fight moment, while the data obtained at the other moments were treated as dependent variables designated as *Y*_baseline_, *Y*_24h_, *Y*_48h_, and *Y*_72h_. In cases of correlation, linear regression was performed to demonstrate the markers temporal evolution.

In order to present hypotheses referring to the sample characteristics, a non-parametric method was used, using the following formula:

$$r_{S}=1-6\overset{n}{\underset{i=1}{\sum }}\frac{d_{i}^{2}}{n\left(n^{2}-1\right)}.$$

The expression above take into account pairs of variables and the difference between the two ranks of {*X*_i_, *Y*_i_}.

Thus, from the calculated *r*_S_ values, the following hypothesis tests were performed to investigate correlation between variables in the population (Kraemer, 1973; Kruskal, 1978).

Hypothesis Test 1

- **Null Hypothesis H**_**0**_: *ρ* = 0. There is no correlation between the population variables *X* and *Y*;
- **Alternative Hypothesis H**_**1**_: *ρ* ≠ 0. There is correlation between the population variables *X* and *Y*.

Linear Regression

The linear regression problem consists into determining a in the matrix equation

Y=Xa+e,

where ***Y*** ≡ response vector, ***X*** ≡ design matrix, ***a*** ≡ vector of regression parameters, and ***e*** ≡ error vector.

The assumed model in the linear adjustment in the used parameters was the second-degree polynomial, by the following equation:

$$Y=a_{1}+a_{2}X+a_{3}X^{2}$$

Above, the adjustment parameters in the fit are {*a*_1_, *a*_2_, *a*_3_}.

The *least-squares method* is able to determine the set of parameters {*a*_1_, *a*_2_, *a*_3_} through the matrix equation (Spearman, 1904). The distribution of residuals in the linear regression is of interest because it allows to evaluate if the variance in the adjustment approaches the minimum for the selected linear estimator. If this distribution is normal, assumptions of the Gauss-Markov theorem are satisfied and the parameters estimation will be the best linear unbiased possible (Scheffe, 1999). A highly efficient test to evaluate if the distribution is normal – available in the Maple System (Char et al., 1983) used in this analysis – is the Shapiro-Wilk. These test hypotheses are.

Hypothesis Test 2

- **H**_**0**_: The residuals of the fit follow a normal distribution.
- **H**_**1**_: The residuals of the fit do not follow a normal distribution.

In this proposal, the Chi-Square goodness of fit test (Char et al., 1983) meets the proposed needs and was applied in combination with the Shapiro-Wilk test. The results of both took into account the level of significance *α* = 0.05. The goodness of fit test hypotheses are presented below.

Hypothesis Test 3

- **H**_**0**_: The calculated observables from the data fitting do not differ from the actual observables.
- **H**_**1**_: The calculated observables from the data fitting differ from the actual observables.

## Results

**Figure 2:**
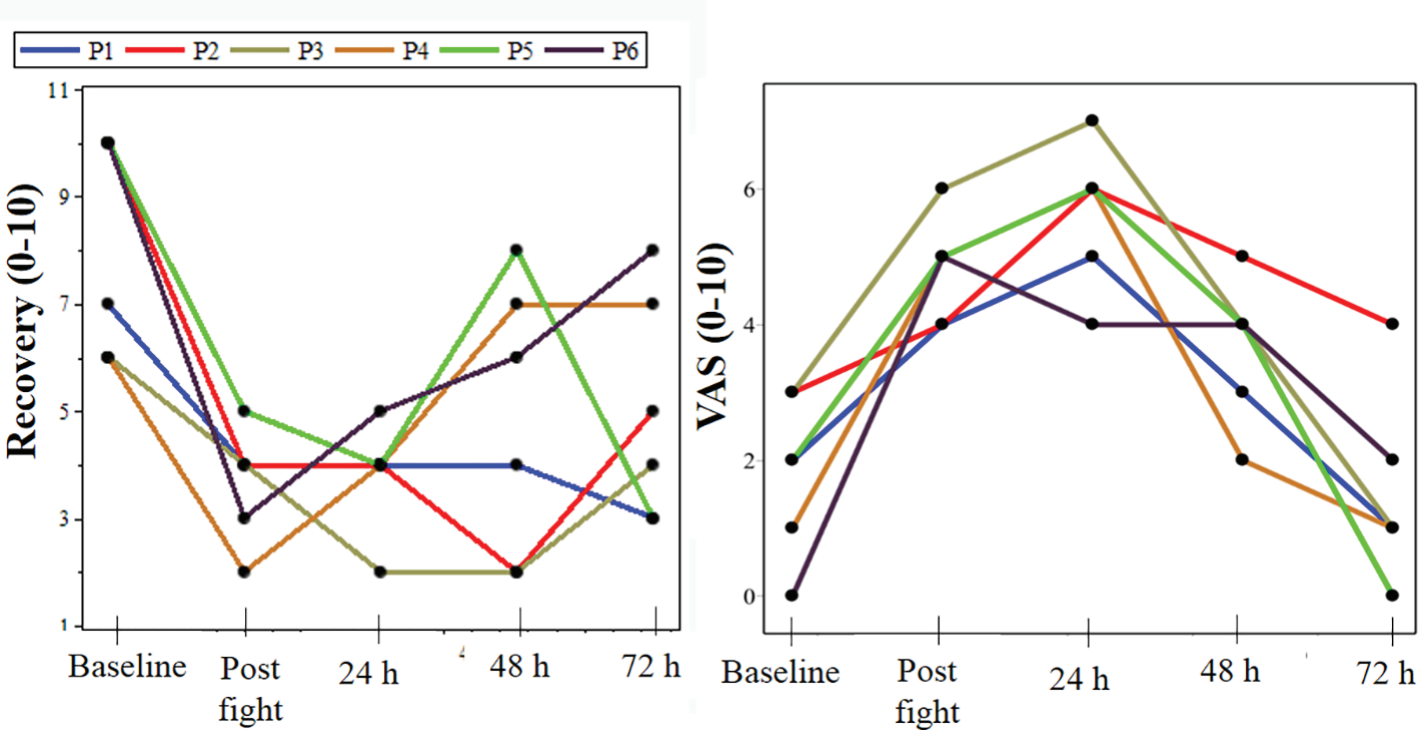
Clinical outcomes, represented by pain and perception of recovery. h, hours; P, parathlete; parathletes I and II were world champions in their respective categories; VAS, analogic visual scale; U/L, units per liter.

**Figure 3:**
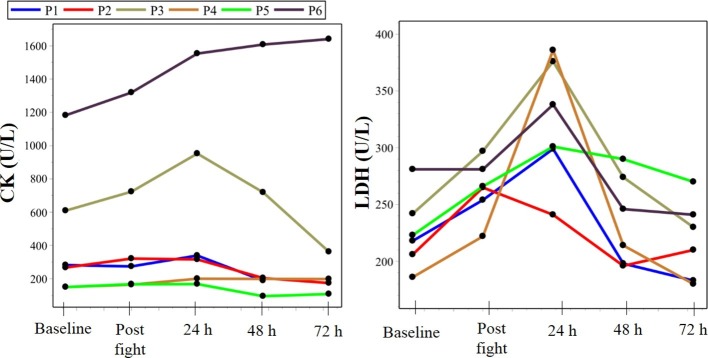
Biochemical markers, represented by creatine kinase (CK) and lactate dehydrogenase (LDH). CK, creatina quinase; LDH, lactate dehydrogenase; P, parathlete. Parathletes I and II were world champions in their respective categories.

**Figure 4:**
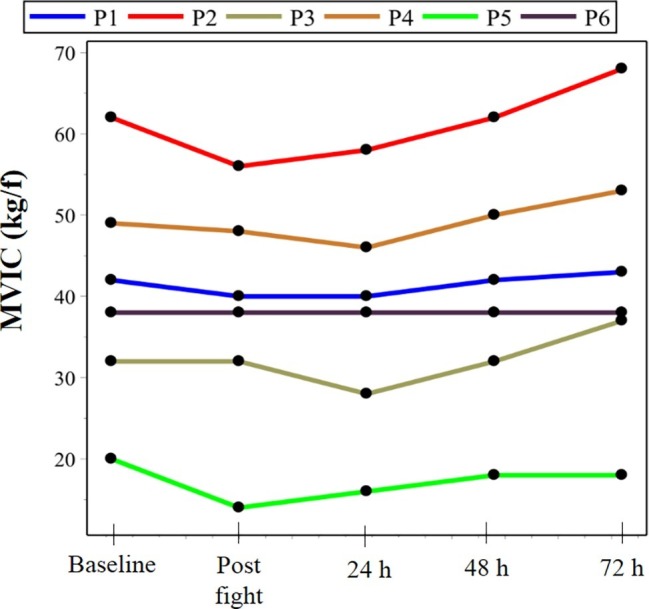
Functional outcomes, represented by isometric strength analysis. MVIC, maximum voluntary isometric contraction; P, parathlete. Parathletes I and II were world champions in their respective categories.

The anthropometric characteristics of the participants are presented in Table 1. Figures 2–4 present the values of the analyzed endpoints of pain, recovery, creatine kinase, lactate dehydrogenase, and strength, respectively.

The peak markers of muscle damage, CK, and LDH occurred between 24 and 48 h after the simulated fight. With regard to CK values, the baseline moment observed were above the reference value (196 IU/L), suggesting that the participants were already in a possible state of muscle injury.

Muscle pain peaked in 24 h. However, the participants reported a reduced pain score, similar to baseline, at 72 h post exercise.

The perception of recovery reduced after the fight, with values that remained low until the 72 h moment. Significant

With respect to strength, there were no significant losses in muscle function between the baseline condition and the moments following the simulated fight.

Table 2 shows the Spearman correlation values obtained from the sample and the limit values for acceptance or rejection at the level of significance *α* = 0.05.

**Table 2:** Spearman correlation between the studied markers.

*M*	Baseline (*Y*_baseline_)			24 h (*Y*_24h_)			48 h (*Y*_48h_)			72 h (*Y*_72h_)		
	*r*_S_	*r*_C_	Res	*r*_S_	*r*_C_	*R*es	*r*_S_	*r*_C_	Res	*r*_S_	*r*_C_	Res
Pain	0.043	0.866	A	0.850	1.000	A	0.050	1.000	A	0.250	1.000	A
Recovery	0.629	0.866	A	0.350	1.000	A	0.325	1.000	A	0.575	1.000	A
CK	0.929	0.866	R	0.886	0.866	R	0.886	0.866	R	0.600	0.866	A
LDH	0.886	0.866	R	0.086	0.866	A	0.600	0.866	A	0.771	0.866	A
MVIC	1.000	0.866	R	1.000	0.866	R	1.000	0.866	R	1.000	0.866	R

*All the coefficients are calculated in relation to the postfight, considered as an independent variable (X) in this analysis. The other observables are considered dependent (Y). The values used in the calculations were extracted from Tables 3, 4 for the set of parathletes I, II, III, IV, V, and VI. The null hypothesis is rejected when p is less than 0.05, which corresponds to the inequality r_S_ > r_C_. M, marker; r_C_, critical coefficient; Res, result; A, accepted; R, rejected*.

The analyses showed a correlation between the variables (*ρ* = 0), CK, LDH, and strength. In the case of LDH indicators, only the observable Y_baseline_ showed no evidence of a significant relationship with the independent variable *X* (post). Although there is statistical evidence in favor of the null hypothesis in the case of the CK indicator between ***Y***_72h_ and ***X***, the others point in favor of the alternative hypothesis.

**Figure 5:**
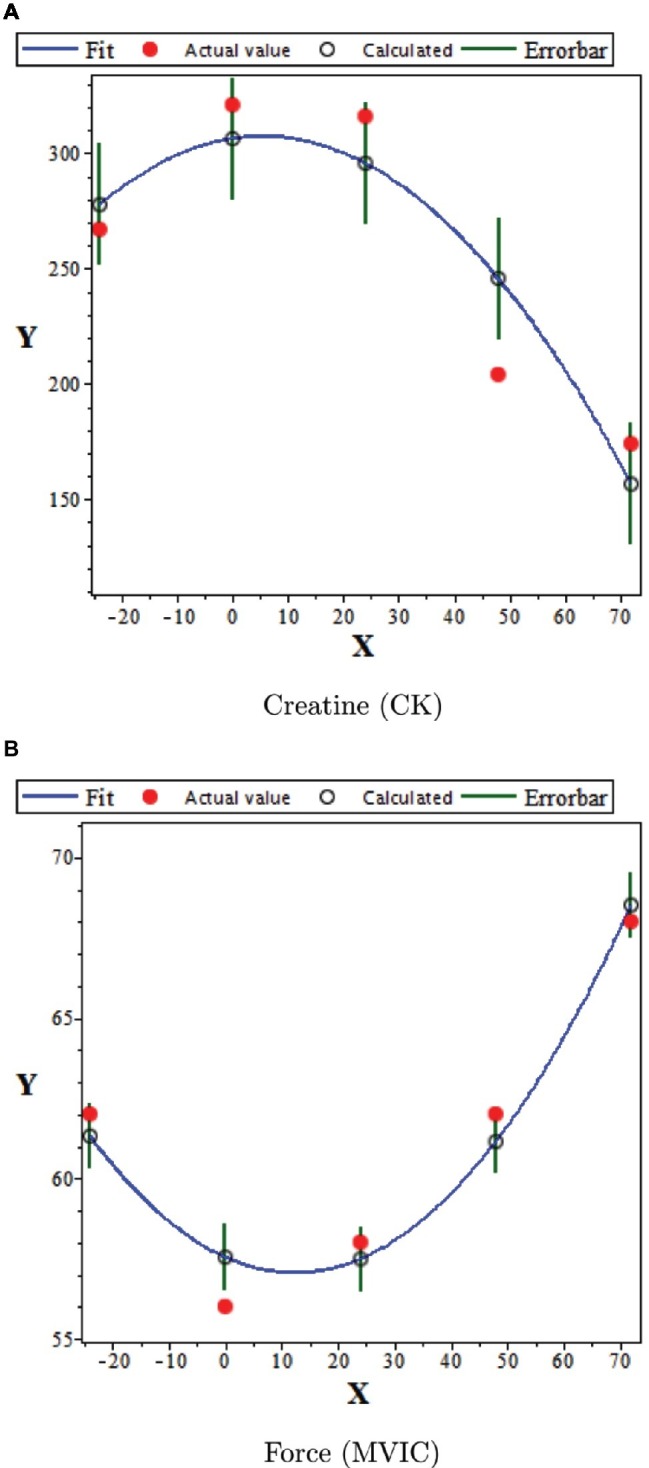
Linear regression for creatine and maximal voluntary isometric contractions. Typical polynomial fit for the time evolution of measurements for creatine **(A)** and maximal voluntary isometric contractions **(B)**. This graph employs the data available in Table 4 for parathlete II. The model for the linear regression is *Y* = *a*_1_ + *a*_2_*X* + *a*_3_*X*^2^. Here, the dependent observable *Y* is the measure of CK or MVIC and the dependent variable *X* is the time in hours. The error bars correspond to the standard deviation of the residuals (RMSE). The graphical representations for the other parathletes are equivalent to these.

Results of the linear regression using the polynomial model are presented below in Figure 5. In these two graphs, the Parathlete II data available in Table 2 were used. The corresponding graphical representations for the other parathletes are equivalent and so are not shown. Keeping the set of independent variables as ***X***, the horizontal axis of the graphs represents the instants at which the indicators were obtained; and these have a delay of 24 h. However, the vertical axes represent the observables of a given marker, assigned as ***Y***. The respective linear regressions analyses are presented in Tables 3, 4.

**Table 3:** Normality tests and goodness-of-fit for the linear regression of the marker CK (**Y**) as a function ofwe (**X**).

Parathlete	Shapiro-Wilk		Goodness-of-fit		
	*p*	Result	*𝟀*2	*p*	Result
I	0.930	Accepted	11.340	0.023	Rejected
II	0.944	Accepted	8.957	0.062	Accepted
III	0.904	Accepted	9.226	0.056	Accepted
IV	0.266	Accepted	400.554	0.000	Rejected
V	0.422	Accepted	0.281	0.991	Accepted
VI	0.865	Accepted	9.956	0.041	Rejected

**Table 4:** Normality tests and goodness-of-fit for the linear regression for MVIC (***Y***) marker as a function of time (***X***).

Parathlete	Shapiro-Wilk		Goodness-of-fit		
	*p*	Result	*𝟀*2	*p*	Result
I	0.238	Accepted	0.025	1.000	Accepted
II	0.238	Accepted	0.070	0.999	Accepted
III	0.803	Accepted	0.255	0.992	Accepted
IV	0.311	Accepted	0.059	1.000	Accepted
V	0.663	Accepted	0.627	0.960	Accepted
VI	0.533	Accepted	0.520	0.720	Accepted

## Discussion

The present study aimed to explore the immediate and delayed physiological responses triggered by combat in Brazilian jiu-jitsu parathletes. The main findings show that the CK and LDH activity in high-performance parathletes was superior and the athletes reported lower muscle pain. The fight did not influence isometric muscular strength levels. Regarding the delayed effects, it was verified peak pain, CK, LDH, and decreased perception of recovery in 24 h. However, within 72 h after the fight, all values had recovered, close to baseline levels.

On the other hand, it has been observed that, in individuals with sedentary deficiency, these values are higher (Hruby and Hu, 2015; Andrea et al., 2018). For this, sport is preventive management since obesity is directly related to high levels of comorbidity, chronic pathologies, psychosocial disorders, and mortality (Coswig et al., 2013; Karjoo, 2018; Kim et al., 2019).

With regard to the training time of the participants, a much higher experience time was observed in the professional parathletes. It is believed that this fact could substantiate the evidence of chronic injury in muscle tissue, in response to inadequate periodization over the years, which would explain the marked CK levels height even in basal conditions in these athletes, since they have reported not adopting an adequate periodization program. In this regard, the study of Adams and Kirkby (2001) addressed the several consequences in body systems due to overtraining, which include high concentrations of CK in individuals who accumulate many hours of training inadequately. After the simulated fight, CK increased in all participants, in the moment 24 h. This fact supports the initial hypothesis that the effort implemented during the practice of Brazilian jiu-jitsu is enough to alter the homeostasis of the systems in the short term. Similar outcomes related to CK behavior after Brazilian jiu-jitsu matches have demonstrated similar results to the present study (Andreato et al., 2015; Detanico et al., 2015; Branco et al., 2016; Fonseca et al., 2016). In addition, it was observed that the recovery of baseline concentration levels occurred at 72 h post-fight. These data also corroborate other studies (Andreato et al., 2015; Branco et al., 2016; Fonseca et al., 2016) in demonstrating that the intensity and volume adopted in this sport modality, when respected, are not sufficient to cause deleterious effects in the long term.

In contrast, some studies (Warren et al., 1999; Morton et al., 2005; Detanico et al., 2015; Prendergasta et al., 2016) state that the actual mechanisms involved in CK alterations are unclear and question whether CK levels reliably assess the accuracy of muscle damage. Furthermore, these studies suggest that the measurement of maximum voluntary isometric contraction is a more relevant parameter (Stanley et al., 2012; Erkan et al., 2015). In this regard, the outcomes related to maximal isometric strength, recorded in the present study, did not demonstrate a statistically significant difference between moments. This finding, associated with the findings of CK activity, supports the ideas presented on the fact that Brazilian jiu-jitsu practice does not cause a significant decline in long-term homeostasis of the body system.

In addition, regarding the isometric muscle strength-related outcomes, the highest values found in high-performance athletes present similar results to other studies which compared the strength level between high-performance athletes and amateurs (Aboodarda et al., 2018) and are observed due to the superior time of physical training. In agreement, a study that investigated Brazilian jiu-jitsu athletes found similar outcomes for strength, with higher levels in professional athletes when compared to amateurs. These findings are essential to assist trainers and athletes in understanding the metabolic demands in jiu-jitsu, acting as a parameter to monitor adaptation and performance during periodized training (Silva et al., 2015).

The perception of recovery has been described as an important tool to evaluate responses to exercise in the adaptive process (Machado et al., 2017), where improvement in recovery perception seems to be directly related to the subsequent performance in sports practice. In this respect, Andreato et al. (2017) suggest that there is a contribution of the psychological mechanism and that high-performance athletes tend to perform better when they believe in the importance of recovery. In addition, higher performance and athletic experience seem to impact less sensitivity to pain and recovery.

With regard to the observed correlation outcomes, only the CK and MVIC indicators justify attribution over the alternative hypothesis –i.e., there is a correlation between the training (moment post-fight) and the posterior moments. On this regard, it is reiterated the fact that correlations quantify the association between the studied markers as a result of the training performed. In contrast, no significant correlations were found between pain, recovery, and handgrip × CK and LDH in post, 24, 48, and 72 h-post. This information is relevant for the association of the discussed markers and serves as a parameter for appropriate periodization prescription, since they relate to the levels of clinical, metabolic, and functional recovery of the subjects in question.

Moreover, the graphical analysis obtained by the linear regression shows a consonance between the calculated values and those observed for the MVIC, as shown in Figure 2. In fact, for all the athletes, the calculated data sample can be considered as corresponding to those observed. The same is not apparent in relation to the temporal dependence of CK measures, since the goodness of fit was not acceptable for athletes I, IV, and VI. Still, for the complexity of a study like this, agreement of half the sample suggests that the polynomial adjustment of the second degree can be proposed as a functional model for the temporal evolution of both indicators, as verified in the exposed data.

Regarding the isometric palmar grip strength outcomes, Andreato et al. (2011) performed the same analysis with Brazilian jiu-jitsu athletes. The outcomes showed mean values of 43.7 ± 4.8 kgf. This result is similar to those reported in the present study. Such similarity demonstrates that the incapacity caused by amputation or visual impairment does not characterize limiting factors that compromise the strength of the upper limbs, highly recruited to perform this sporting modality. In addition, these findings are encouraging and should serve as a motivation for practitioners of Brazilian Jiu-Jitsu paradesports, since strength characterizes basic physical ability, being responsible for the good functionality of the subject.

In summary, the presented results provide us with support to infer that the whole strategy used in this analysis serves the research purpose. In this sense, the data demonstrate important correlations between clinical, metabolic, and functional parameters in response to the Brazilian jiu-jitsu paradesportivo practice. In addition, the proposed model for the CK and MVIC indicators presents a logical temporal reasoning supported by reference scientific literature that indicates reliable outcomes and encourages the improvement and use of this type of analysis in the human physiology research field.

To the knowledge of the authors, this is the first study to verify clinical, functional, and muscle damage marker outcomes in response to Brazilian jiu-jitsu paradesportivo practice. This may be due to the possibility of large variations between the types of disabilities, which may influence the heterogeneity of the assessed group associated with the logistical difficulty mentioned above in gathering a high number of individuals with the same type of disability. In addition, this difficulty characterizes a limitation. However, it is necessary to consider the theme and to understand the kinetic behavior in response to the practice of this sport modality, to assist the scientific and clinical community in the management of specific actions that aid athletic performance and lower the incidence of injuries in this particular population.

The current study presents strengths. First, it was elaborated with high methodological quality. Second, the procedures were carried out in a field setting, identical to that used in competitive combat. In addition, the presented outcomes are unprecedented and constitute intrinsic potential under new perspectives and parameters related to the parasport. It is pertinent that future studies address the analysis of other biochemical markers such as hormonal rate and cytokines as well as the application of specific recovery techniques based on the observed physiological responses in order to measure possible differences in physiological parameters after the fight.

## Conclusion

The findings of this study demonstrated the results of biochemical markers related to muscle damage, after a fight, in professional and amateur Brazilian jiu-jitsu parathletes. For this, peak values of the analyzed variables were recorded in 24 h. However, at 72 h, the values returned to levels close to baseline. It was also observed that there were no deleterious effects on muscle function after the fight. The presented outcomes provide parameters and suggest a safe scenario based on the intensity and volume adopted in this parasport modality.

## Ethics Statement

Participants signed a free and informed consent form, agreeing to participate in it. In addition, the study was approved by the ethics and research committee involving human subjects of the Federal University of Mato Grosso (UFMT).

## Author Contributions

JL designed the study, conducted the analyses, and wrote the manuscript. PA, AA, and LG assisted in the acquisition, analysis, and interpretation of data, and reviewed and edited the article. CA and AN made substantial contributions including conception and design of the study, and a critical revision of the article. All authors read and approved the final manuscript.

### Conflict of Interest Statement

The authors declare that the research was conducted in the absence of any commercial or financial relationships that could be construed as a potential conflict of interest.
